# *Para*-Hydroxycinnamic Acid Mitigates Senescence and Inflammaging in Human Skin Models

**DOI:** 10.3390/ijms25158153

**Published:** 2024-07-26

**Authors:** Christina Yan Ru Tan, Malgorzata Morenc, Melina Setiawan, Zen Zhi Yan Lim, Ai Ling Soon, John C. Bierman, Laura Vires, Timothy Laughlin, Yvonne M. DeAngelis, Holly Rovito, Bradley B. Jarrold, Thi Quynh Ngoc Nguyen, John Soon Yew Lim, Olivia Kent, Arto Määttä, Adam M. Benham, Timothy J. Hawkins, Xin Er Lee, Matthew C. Ehrman, John E. Oblong, Oliver Dreesen, Sophie Bellanger

**Affiliations:** 1 A*STAR Skin Research Labs (A*SRL), Skin Research Institute of Singapore (SRIS), Agency for Science, Technology and Research (A*STAR), 8A Biomedical Grove, #06-06 Immunos, Singapore 138648, Singapore; christina_tan@asrl.a-star.edu.sg (C.Y.R.T.); melina_setiawan@asrl.a-star.edu.sg (M.S.); zen_lim@asrl.a-star.edu.sg (Z.Z.Y.L.); ailingsoon@ntu.edu.sg (A.L.S.); anna_nguyen@asrl.a-star.edu.sg (T.Q.N.N.); john_lim@asrl.a-star.edu.sg (J.S.Y.L.); lee_xin_er@asrl.a-star.edu.sg (X.E.L.); oliver_dreesen@asrl.a-star.edu.sg (O.D.); 2 The Procter & Gamble Company, Mason, OH 45040, USA; bierman.jc@pg.com (J.C.B.); vires.l@pg.com (L.V.); laughlin.lt@pg.com (T.L.); deangelis.ym@pg.com (Y.M.D.); rovito.ha@pg.com (H.R.); jarrold.bb@pg.com (B.B.J.); oblong.je@pg.com (J.E.O.); 3 Department of Biosciences, Durham University, South Road, Durham DH1 3LE, UK; olivia.v.kent@durham.ac.uk (O.K.); arto.maatta@durham.ac.uk (A.M.); adam.benham@durham.ac.uk (A.M.B.); t.j.hawkins@durham.ac.uk (T.J.H.); 4 Procter & Gamble International Operations SA SG Branch, 70 Biopolis Street, Singapore 138547, Singapore; ehrman.mc@pg.com

**Keywords:** aging, senescence, inflammation, oxidative stress, *para*-coumaric acid, *para*-hydroxycinnamic acid, keratinocyte, inflammaging, DNA damage, DNA repair

## Abstract

*Para*-hydroxycinnamic acid (pHCA) is one of the most abundant naturally occurring hydroxycinnamic acids, a class of chemistries known for their antioxidant properties. In this study, we evaluated the impact of pHCA on different parameters of skin aging in in vitro skin models after H_2_O_2_ and UV exposure. These parameters include keratinocyte senescence and differentiation, inflammation, and energy metabolism, as well as the underlying molecular mechanisms. Here we demonstrate that pHCA prevents oxidative stress-induced premature senescence of human primary keratinocytes in both 2D and 3D skin models, while improving clonogenicity in 2D. As aging is linked to inflammation, referred to as inflammaging, we analyzed the release of IL-6, IL-8, and PGE_2_, known to be associated with senescence. All of them were downregulated by pHCA in both normal and oxidative stress conditions. Mechanistically, DNA damage induced by oxidative stress is prevented by pHCA, while pHCA also exerts a positive effect on the mitochondrial and glycolytic functions under stress. Altogether, these results highlight the protective effects of pHCA against inflammaging, and importantly, help to elucidate its potential mechanisms of action.

## 1. Introduction

All of our organs are subjected to the effects of time, known as chronological aging (or intrinsic aging), characterized by specific morphological changes of both the dermis and the epidermis. Modifications include the loss of skin homeostasis with overall skin thinning, the appearance of wrinkles and fine lines, the loss of firmness and elasticity, dryness, pigmented spots, and slower wound healing. In addition, aging in skin is accelerated by external stimuli (extrinsic aging), such as pollution generating oxidative stress or UV exposure (photoaging) [1].

UV radiation (UVR) exerts some positive effects on systemic homeostasis by facilitating the synthesis of biologically active molecules, including the well-known production of vitamin D3. In addition, UVR also influences the production of neuroendocrine and immune factors, which can either be released into the bloodstream, or stimulate nerve endings in the skin. This, in turn, activates the brain, endocrine, and immune systems, contributing, for example, to mood regulation through the synthesis of serotonin and endorphins in the central nervous system, or to anti-inflammatory responses through the synthesis of POMC (pro-opiomelanocortin)-derived peptides and eventually corticosteroid production [2]. However, UVR is better known for its negative effects on skin, where prolonged and repeated exposure not only accelerates skin aging, but also increases the risk of developing skin cancers such as basal cell carcinoma (BCC), squamous cell carcinoma (SCC), and melanoma.

While UVB has a direct mutagenic effect where radiation is absorbed by DNA to generate cyclobutane pyrimidine dimers (CPD) lesions followed by double-strand breaks in epidermal cells, there is evidence of a secondary indirect accumulation of reactive oxygen species (ROS) via the activation of catalase, cyclooxygenase, and NADPH oxidase [3]. UVA radiation, which has a better penetration than UVB and reaches the reticular dermis, directly mediates oxidation reactions through excited photosensitizers, while a secondary production of ROS also occurs as a delayed process following exposure [3]. Oxidative stress occurs when an imbalance arises between the production of ROS and the detoxification of these highly reactive intermediates by ROS scavengers naturally present in cells. At the molecular level, oxidative stress or UV exposure leads to various cellular damages affecting DNA, but also proteins, lipids and mitochondria, leading to accelerated human primary keratinocyte (HPK) differentiation, senescence, and inflammation [4,5].

Senescence is characterized by an irreversible cell cycle arrest driven by the increase in p53/p21 or p16/Rb. In addition, lamin B1, an intermediate filament protein and component of the nuclear envelope, is downregulated in senescent cells, thereby serving as a biomarker of senescence, including in HPKs [6,7]. Senescent cells are viable and still metabolically active. They secrete a cocktail of factors known as the senescence-associated secretory phenotype (SASP) [8,9]. SASP molecules include pro-inflammatory cytokines such as interleukin 6 and 8 (IL-6 and IL-8), proteases such as metalloproteinases (i.e., MMPs which degrade collagen, resulting in the loss of both skin firmness and plumpness, thereby favoring wrinkles), and growth factors. In addition, prostaglandin E_2_ (PGE_2_, a lipid mediator derived from arachidonic acid metabolism after the action of cyclooxygenase enzymes COX-1 and 2) can also be secreted by senescent human keratinocytes, which could be linked to carcinogenesis [10].

*Para*-hydroxycinnamic acid (pHCA, aka *p*-coumaric acid or 4-hydroxycinnamic acid) is a plant-derived secondary phenolic metabolite, and one of the most abundant members of the hydroxycinnamic acid family, which recently garnered attention for its potential effects on skin health [11,12,13]. Secondary polyphenolic acids are found in various plant sources (including edible ones such as cereals, mushrooms, fruits, vegetables, and some beverages like wine, tea, and coffee), where they help ameliorate the detrimental consequences of UV exposure and environmental stress. Indeed, in addition to exhibiting a sun protection factor (SPF) due to their capacity to absorb UV (also known as sunscreen effect) [14], some natural polyphenols act as ROS scavengers, accounting for their antioxidant power [15,16], while others improve DNA repair (such as green tea polyphenols) and limit inflammation induced by oxidative stress in mammals after UV exposure (in in vitro and in vivo mouse and human models) [17,18]. For instance, green tea polyphenols such as catechins or flavanols, can reduce COX-2 and prostaglandin metabolites (e.g., PGE_2_) induced by UVB, known to exert a crucial role in inflammatory skin disorders [19]. In addition, green tea polyphenols boost the repair of CPD lesions through the nucleotide excision repair (NER) mechanism [19]. Regarding pHCA specifically, it prevents oxidative stress-induced cellular senescence in murine chondrocytes, as well as IL-1β-induced inflammation, and may have skin brightening properties [20,21]. However, the impact of pHCA on cellular senescence in human skin cells has not been explored.

This study focused on examining the impact of pHCA on aging in human skin, particularly senescence and inflammation. The research also aimed to uncover potential new upstream mechanisms through which pHCA might act, such as the modulation of premature differentiation, DNA damage/repair, as well as mitochondrial and glycolytic functions. To achieve this, we use both 2D keratinocyte cultures and 3D organotypic skin models (including epidermal and full-thickness models) subjected to UV exposure or oxidative stress (H_2_O_2_ exposure). The keratinocyte fate was then investigated in both the absence and presence of pHCA to determine cellular responses and pathways modified. Niacinamide (NAM) was used as a positive control for primary endpoints: cellular senescence, premature differentiation, inflammation, and DNA damage/repair experiments. Here, we demonstrate that pHCA significantly reduces oxidative stress-induced senescence and exerts a strong impact on inflammation while barely preventing HPK premature differentiation. Key possible mechanisms of action identified for pHCA include lower levels of ROS as expected, but also the prevention of DNA damage accumulation (rather than enhanced DNA repair), and improved energy metabolism under stress.

## 2. Results

### 2.1. pHCA Prevents Senescence Induced by Oxidative Stress in 2D HPK Cultures and 3D Organotypic Epidermal Models, While Premature Differentiation Is Barely Prevented

**Figure 1:**
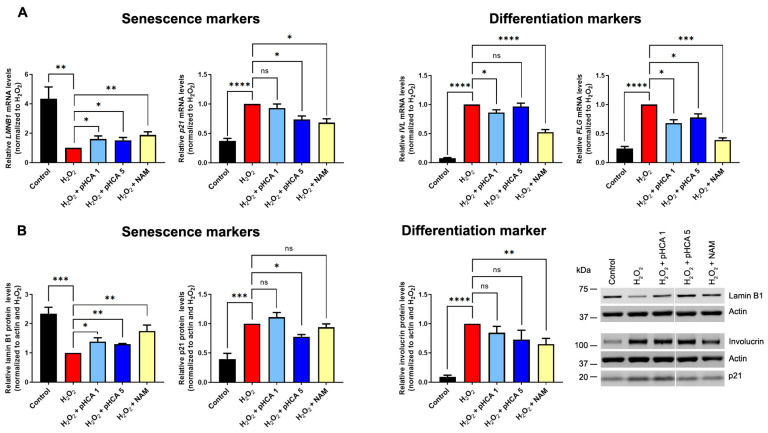
pHCA prevents senescence induced by oxidative stress in 2D HPK cultures, while premature differentiation is barely prevented. (**A**) RT-qPCR analyses after treatment of HPKs with H_2_O_2_ (63 μM) ± pHCA (1 or 5 μM) or NAM (1.5 mM). Statistical analyses: one sample *t*-tests with a hypothetical value of 1, n = 4–7. (**B**) Western blot quantifications after treatment of HPKs as in (**A**). Statistical analyses: one sample *t*-tests with a hypothetical value of 1, n = 3–8. * *p* ≤ 0.05; ** *p* ≤ 0.01; *** *p* ≤ 0.001; **** *p* ≤ 0.0001. A representative Western blot is shown on the right panel. Abbreviations: HPKs, human primary keratinocytes; pHCA, *para*-hydroxycinnamic acid; NAM, niacinamide; RT-qPCR, reverse-transcription quantitative PCR; LMNB1, lamin B1; IVL, involucrin; FLG, filaggrin; ns, not significant.

To investigate the effects of pHCA on oxidative stress in skin, 2D cultures of HPKs were subjected to H_2_O_2_ treatment in the presence or absence of pHCA (two concentrations were tested, 1 μM and 5 μM) or niacinamide (NAM) at 1.5 mM (positive control, known to prevent premature senescence and differentiation induction in similar conditions [5]). Both *LMNB1* (lamin B1) and *p21* mRNAs were analyzed by reverse transcription-quantitative polymerase chain reaction (RT-qPCR) in parallel to the late differentiation markers *FLG* (filaggrin) and *IVL* (involucrin) mRNAs (Figure 1A). As expected, after H_2_O_2_ exposure, *LMNB1* mRNA levels were significantly lower than in control cells, while *p21* levels increased. The pHCA treatment either at 1 μM or 5 μM limited the drop of *LMNB1*, while NAM treatment showed a similar efficiency. The increase in *p21* levels was also partially prevented by NAM and pHCA at 5 μM (Figure 1A). At the protein levels, the results were very comparable, with significant protective effects of both pHCA and NAM on lamin B1 loss, and on p21 increase at the higher pHCA concentration (Figure 1B). The analysis of differentiation markers showed a trivial effect of pHCA as compared to NAM. Indeed, while NAM prevented the premature induction of both late differentiation markers *FLG* and *IVL* at the mRNA level, pHCA only modestly prevented *FLG* induction at both concentrations, and no consistent effect was observed on *IVL* (Figure 1A). At the protein level, while involucrin was downregulated by NAM, the effect of pHCA appeared non-significant (Figure 1B). 

**Figure 2:**
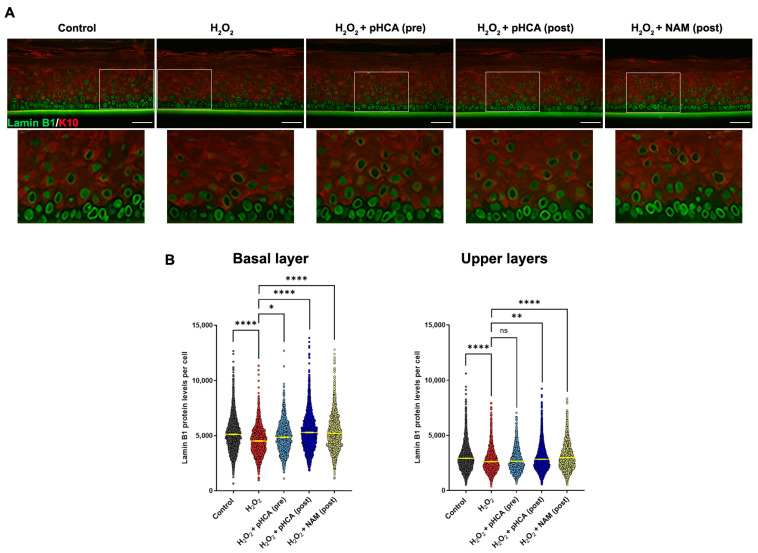
pHCA prevents senescence induced by oxidative stress in 3D organotypic epidermal models. (**A**) Immunofluorescence staining (400×) of 3D epidermal organotypics subjected to H_2_O_2_ challenge, pre- or post-treated with pHCA (K10 in red, Alexa Fluor 568; lamin B1 in green, Alexa Fluor 488). Post-stress treatment with NAM was used as a positive control. Scale bar = 50 µm. For each picture, a magnification is shown (white rectangle). (**B**) Quantifications of lamin B1 levels per cell in K10-negative (basal layer) and in K10-positive (upper layers) cells. Each dot represents one cell. A total of 3301–4466 cells were analyzed per condition (from six replicates). Statistical analyses: one-way ANOVA. * *p* ≤ 0.05; ** *p* ≤ 0.01; **** *p* ≤ 0.0001. Abbreviations: pHCA, *para*-hydroxycinnamic acid; NAM, niacinamide; K10, keratin 10; ns, not significant.

Three-dimensional organotypic epidermal models were used to confirm the senescence prevention observed in 2D culture in a more physiological setup (Figure 2). For this, mature epidermal organotypics were either treated with pHCA (10 μM was used here due to the differences in complexity and architecture as compared to 2D models) for 16 h, before challenge with 125 μM H_2_O_2_ (pre-stress treatment), or treated with pHCA immediately after H_2_O_2_ challenge (post-stress treatment). NAM post-stress treatment was used as a positive control. Organotypics were fixed 6 days after H_2_O_2_ challenge and sectioned before staining for lamin B1 and keratin 10 (Figure 2A), as well as Ki-67. While the Ki-67 staining showed no significant differences in any of the conditions tested (Supplementary Figure S1), as previously published [5], lamin B1 levels significantly decreased after H_2_O_2_ treatment in both the basal and upper layers indicating senescence, while NAM significantly prevented this drop of lamin B1 as we previously published (Figure 2B) [5]. Treatment with pHCA either before or after H_2_O_2_ challenge allowed the maintenance of high levels of lamin B1 (indicating the prevention of senescence), with a better efficiency in basal than upper layers. Interestingly, post-stress treatment with pHCA appeared more efficient than pre-stress treatment in both the basal and upper layers.

### 2.2. pHCA Improves Clonogenicity after Oxidative Stress in 2D Cultures of HPKs

**Figure 3:**
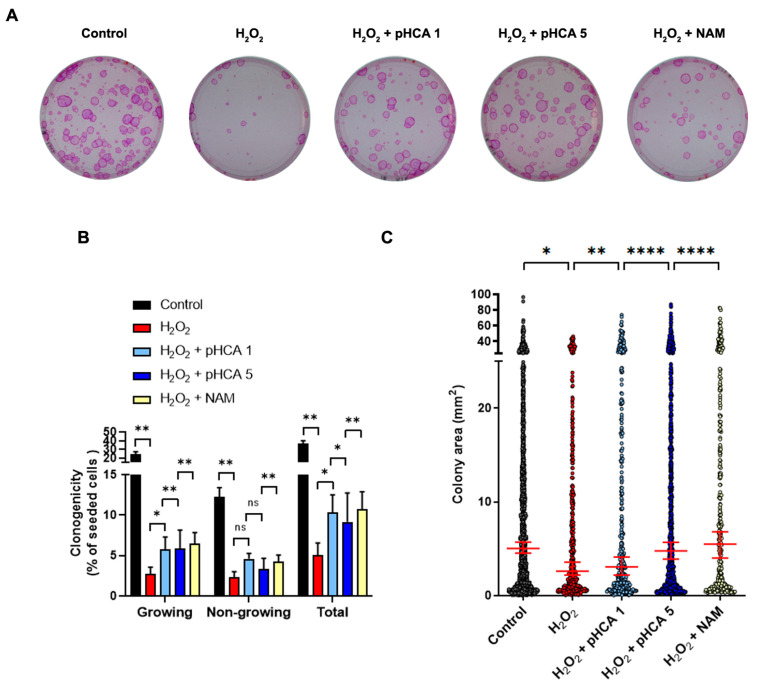
pHCA improves clonogenicity after oxidative stress in 2D cultures of HPKs. (**A**) Clonogenicity tests performed without treatment (Control) or after H_2_O_2_ challenge ± pHCA (1 or 5 μM) or NAM. pHCA and NAM were maintained in the medium for 5 days before seeding the cells for clonogenicity tests. Rhodamine B staining 12 days after seeding is shown. (**B**) Quantification of total, non-growing, and growing colonies shown in (**A**). Statistical analyses: ratio paired *t*-tests, n = 4–6. (**C**) Analyses of colony area. Statistical analyses: one-way ANOVA, n = 324–1643 colonies analyzed per condition. * *p* ≤ 0.05; ** *p* ≤ 0.01; **** *p* ≤ 0.0001. Abbreviations: HPKs, human primary keratinocytes; pHCA, *para*-hydroxycinnamic acid; NAM, niacinamide; ns, not significant.

The effects of pHCA (1 μM and 5 μM) on oxidative stress were verified using clonogenic assays to assess the HPK regenerative capacity. Two-dimensional cultures of HPKs were treated with H_2_O_2_ for 30 min in the presence or absence of pHCA or NAM, which then remained in the media for 5 days before clonogenic assays (pHCA and NAM were absent during the 12 days of clonogenic expansion). As expected, the H_2_O_2_ challenge induced a massive loss of the HPK proliferative capacity, as evidenced by Rhodamine B staining, revealing decreased colony number and size (Figure 3A). The number of colonies and the area of each colony were quantified using custom-made macros (Supplementary Materials and Methods) showing a total colony forming efficiency of 5.0% after oxidative stress as compared to 36.6% for the control population (Figure 3B, compare the black and red bars in “Total”). As expected from the senescence results, both pHCA- and NAM-treated populations showed an improved total clonogenic capacity by about 2-fold. Regarding the proportion of “Non-growing” versus “Growing” colonies, the ratio between differentiated (paraclones, “Non-growing”) and proliferative colonies (holoclones + meroclones, “Growing”) increased from 0.50 for the control to 0.86 after H_2_O_2_ treatment (i.e., less colonies are attached and those attached are more differentiated). While the NAM-treated population showed a ratio of 0.65, it was 0.80 for 1 μM pHCA but decreased to 0.61 for 5 μM pHCA (i.e., less differentiated colonies for the higher concentration—Figure 3B), consistent with the better repression of p21 observed at the higher pHCA concentration. Regarding the area of the colonies, as anticipated, H_2_O_2_ challenge led to smaller colonies, while both NAM and pHCA significantly improved the mean colony area. Again, a better effect was observed at 5 μM for pHCA, probably due to the lower number of differentiated (“Non-growing”) colonies obtained with this concentration (Figure 3C). Altogether, these data show that pHCA preserves the regenerative capacity of HPKs after oxidative stress, likely driven primarily by senescence prevention rather than premature differentiation inhibition, although some subtle effects on differentiation are visible in both Figure 1 and Figure 3.

### 2.3. pHCA Reduces ROS Levels in Keratinocytes

**Figure 4:**
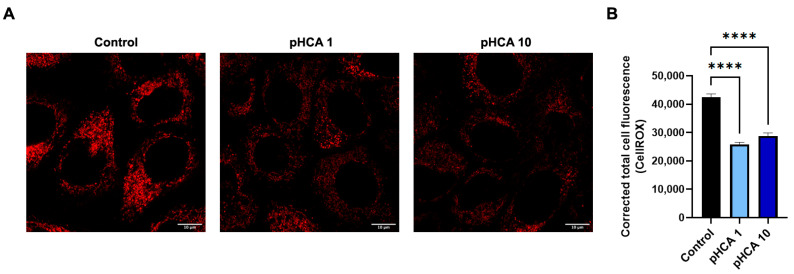
pHCA reduces ROS levels in keratinocytes. (**A**) CellROX staining after treatment of HaCaT cells with 1 or 10 μM pHCA as indicated. Scale bar = 10 µm. (**B**) Quantification of CellROX staining. Statistical analyses: two-way ANOVA, n = 55–71 cells analyzed per condition (from triplicates). **** *p* ≤ 0.0001. Abbreviation: pHCA, *para*-hydroxycinnamic acid.

To evaluate potential upstream mechanisms of action, the cellular oxidative stress was measured via CellROX using immortalized HaCaT human keratinocytes treated with either 1 μM or 10 μM pHCA. The ROS fluorescence intensity was imaged after 1 h by confocal microscopy and quantified. The fluorescent signal was found to cluster around the nucleus and was relatively uniformly spread within the cytoplasm (Figure 4A). Treatment with pHCA at both concentrations significantly reduced ROS cellular levels (Figure 4A,B). 

### 2.4. pHCA Prevents H_2_O_2_-Induced DNA Damage

**Figure 5:**
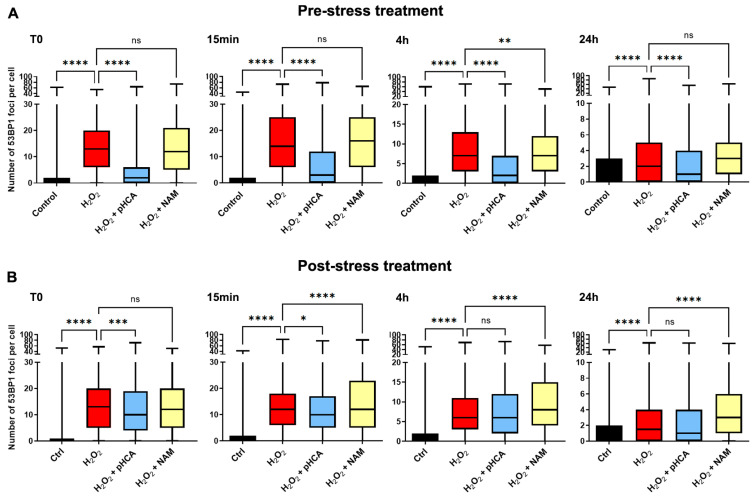
pHCA prevents H_2_O_2_-induced DNA damage. (**A**) Pre-stress treatment: HPKs were treated with pHCA (1 μM) or NAM for 24 h prior to challenge with 63 μM H_2_O_2_ (pHCA and NAM were not added during, nor after, H_2_O_2_ treatment). (**B**) Post-stress treatment: HPKs were treated with 63 μM H_2_O_2_, while pHCA or NAM were added during and after H_2_O_2_ challenge. The cells were fixed at the indicated time points after H_2_O_2_ treatment, and the number of 53BP1 foci per cell was quantified using immunofluorescence microscopy. Statistical analyses: one-way ANOVA, n = 1116–1685 cells analyzed per condition (from four replicates). * *p* ≤ 0.05; ** *p* ≤ 0.01; *** *p* ≤ 0.001; **** *p* ≤ 0.0001. Abbreviations: HPKs, human primary keratinocytes; pHCA, *para*-hydroxycinnamic acid; NAM, niacinamide; min, minutes; h, hours; ns, not significant.

To further analyze the mechanisms of action of pHCA, HPKs were treated for 24 h before H_2_O_2_ challenge (pre-stress treatment) or during and after H_2_O_2_ challenge (post-stress treatment) with pHCA (1 μM, only one concentration was chosen since 1 μM and 5 μM gave similar senescence, differentiation, and clonogenicity results) or NAM. The DNA damage response was analyzed at different time points after H_2_O_2_ treatment using anti-53BP1 immunofluorescence staining to visualize double-strand break foci under repair, known to appear as dots (Supplementary Figures S2 and S3 for pictures, and Figure 5 for dot quantification results).

As expected, in the pre-stress treatment experiment, H_2_O_2_ exposure led to an immediate re-localization of 53BP1 to distinct foci (T0). While NAM-treated cells showed a similar profile, strikingly, a very low response was observed in pHCA-treated cells, indicating much lower levels of DNA damage (Figure 5A and Supplementary Figure S2). The 15 min and 4 h time points showed profiles comparable to T0, although a lower foci number was observed at 4 h in all samples (expected when the lesions resolve—compare scales between the 15 min and 4 h graphs). By 24 h, most of the foci cleared in all samples, while the foci number in pHCA-treated cells was still significantly lower than in the H_2_O_2_ and H_2_O_2_ + NAM-treated samples.

Post-stress treated samples all showed very similar profiles of high DNA repair response at T0, indicating elevated levels of damage regardless of the compound used (Figure 5B and Supplementary Figure S3). Of note, pHCA showed a significantly lower number of foci, presumably due to the presence of pHCA during the H_2_O_2_ challenge. From 15 min onwards and until the end of the experiment, while 53BP1 foci progressively cleared in all samples, NAM-treated cells consistently showed significantly higher numbers of DNA repair foci than the control and pHCA-treated cells, confirming improved/prolonged DNA repair response in the presence of NAM, as previously published [5]. Altogether, these data show that while NAM improves DNA repair, pHCA does not, but instead prevents DNA damage, probably through the prevention of ROS generation.

### 2.5. pHCA Mitigates UV-Induced Inflammatory Response in 2D and 3D In Vitro Models

**Figure 6:**
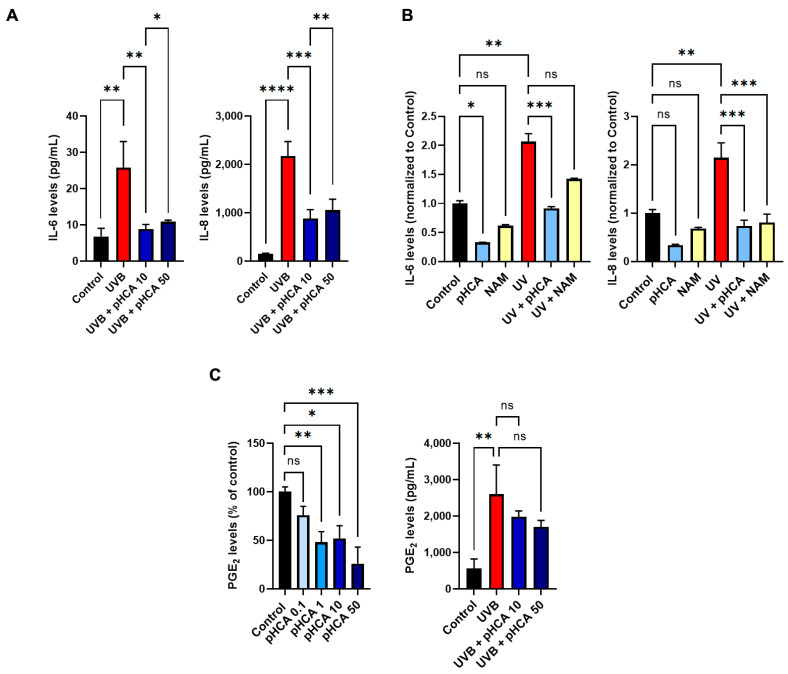
pHCA mitigates UV-induced inflammatory response in 2D and 3D in vitro models. (**A**) IL-6 and IL-8 levels were measured in basal conditions (Control) or after treatment of hTERTs with UVB followed by pHCA treatment (10 or 50 μM). Statistical analyses: one-way ANOVA, n = 4. (**B**) Three-dimensional full-thickness organotypic skin models were pre-treated with pHCA or NAM for 48 h before UV exposure (1.7 J/cm^2^ UVA + 100 mJ/cm^2^ UVB, in the absence of pHCA and NAM), and post-treated with pHCA or NAM for an additional 48 h period. The medium was harvested and subjected to ELISA assays. Statistical analyses: two-way ANOVA, n = 3. (**C**) Left panel: PGE_2_ levels were measured in hTERTs in basal conditions after treatment with increasing doses of pHCA (0.1 to 50 μM). Right panel: hTERTs were treated as in (**A**) and PGE_2_ levels were quantified using HTFR. Statistical analyses: one-way ANOVA, n = 4. * *p* ≤ 0.05; ** *p* ≤ 0.01; *** *p* ≤ 0.001; **** *p* ≤ 0.0001. Abbreviations: pHCA, *para*-hydroxycinnamic acid; NAM, niacinamide; IL, interleukin; PGE_2_, prostaglandin E_2_; HTFR, homogeneous time resolved fluorescence; ELISA, enzyme-linked immunosorbent assay; ns, not significant.

Next, we tested the effect of pHCA treatment on inflammation in both human telomerase reverse transcriptase-immortalized (hTERT) keratinocytes and 3D full-thickness organotypic models. Given that the inflammatory response is more pronounced after UV than H_2_O_2_ treatment in our 3D full-thickness models, we focused on stress induced by UV to study inflammatory markers. hTERT keratinocytes were exposed to 25 mJ/cm^2^ UVB before overnight treatment with pHCA to measure the effect of pHCA on mitigating the UVB-induced release of IL-6 and IL-8 levels. While both IL-6 and IL-8 increased with UV exposure as expected, treatment with pHCA, either at 10 μM or 50 μM, was found to significantly reduce the secreted levels of both inflammatory biomarkers (Figure 6A).

To further evaluate the effect of pHCA on inflammation under more physiological conditions, 3D full-thickness organotypic skin models were treated with pHCA (50 μM was used here due to the presence of the extracellular matrix in the dermis which limits the effective concentration of pHCA reaching HPKs in the epidermis) in basal conditions, or pre-treated with pHCA for 48 h before exposure to 1.7 J/cm^2^ UVA + 100 mJ/cm^2^ UVB (in the absence of pHCA) followed by post-treatment with pHCA for an additional 48 h period. NAM, previously shown to reduce SASP-associated inflammatory cytokines following the same protocol, was used as a positive control [5]. The pHCA treatment demonstrated a reduction in both inflammatory biomarkers in basal and stress conditions, with the highest efficiency observed for IL-8 after UV exposure. Importantly, in all conditions tested, pHCA appeared more efficient than NAM at reducing inflammation (Figure 6B).

PGE_2_ levels were investigated in 2D experiments (hTERT keratinocytes) as its synthesis is also related to senescence. Without stress, a significant dose-dependent reduction in PGE_2_ levels was observed after treatment with 1 μM, 10 μM, and 50 μM pHCA, as compared to the control. After UVB exposure, pHCA was also found to prevent PGE_2_ secretion, although the efficiency appeared lower than for IL-6 and IL-8 and did not reach significance (Figure 6C). 

### 2.6. pHCA Maintains Energy Metabolism

**Figure 7:**
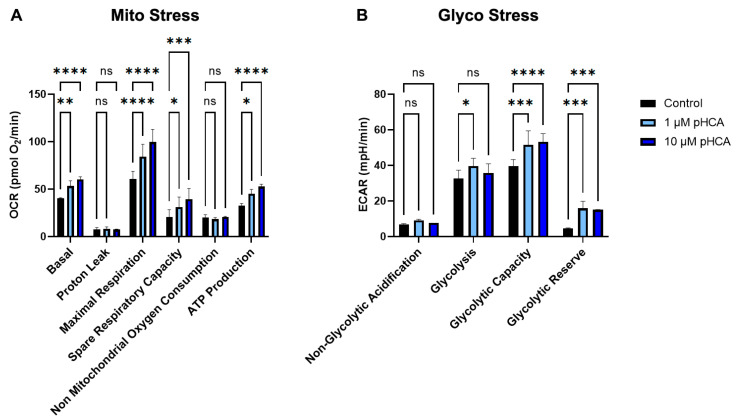
pHCA maintains energy metabolism. (**A**) OCR (OXPHOS) and (**B**) ECAR (glycolysis) measurements in hTERTs treated with 1 or 10 μM pHCA, subjected to Mito Stress and Glyco Stress assays, respectively. Statistical analyses: two-way ANOVA, n = 4. * *p* ≤ 0.05; ** *p* ≤ 0.01; *** *p* ≤ 0.001; **** *p* ≤ 0.0001. Abbreviations: pHCA, *para*-hydroxycinnamic acid; OCR, oxygen consumption rate; OXPHOS, oxidative phosphorylation; ECAR, extracellular acidification rate; min, minute; ns, not significant.

To evaluate the effects of pHCA (1 μM and 10 μM) on the recovery of mitochondrial function after stress, metabolic stress testing was conducted using the Seahorse XL FluxAnalyzer™. The Mito Stress test was used on hTERT keratinocytes treated with pHCA for 24 h, and then sequentially exposed to oligomycin, carbonyl cyanide 4-(trifluoromethoxy) phenylhydrazone (FCCP), rotenone, and antimycin C, as per manufacturer’s instructions. The pHCA treatment demonstrated a significant increase in basal respiration, maximal respiration, spare respiratory capacity, and ATP production, showing protected mitochondrial functions under stress (Figure 7A). For glycolytic stress testing (Glyco Stress), hTERT keratinocytes were treated with pHCA for 24 h, and then sequentially exposed to glucose, oligomycin, and 2-deoxy-D-glucose, as per manufacturer’s instructions. The pHCA treatment was found to significantly increase the glycolytic capacity and the glycolytic reserve (Figure 7B).

## 3. Discussion

Skin aging is associated with cumulative exposure to extrinsic and intrinsic stressors over time, leading to the reduced ability of the skin to cope with stressors, and increasingly pro-inflammatory cellular conditions [22,23]. This inflammatory cascade is termed “inflammaging”, reflecting the various associations between inflammation and the hallmarks of aging, including cellular senescence, altered intercellular communication, mitochondrial dysfunction, and genomic instability [24,25]. Hence, there is a need in the aging field to study bioactive molecules with protective anti-senescence and anti-inflammatory effects, as well as the associated upstream and downstream molecular pathways.

Hydroxycinnamic acids, as potent antioxidants, are good candidates to target senescence and inflammaging in skin [12,13,14,15,26]. Elucidating their biomolecular effects and mechanisms of action could help design and deliver effective bioactive treatments for photoaging and stress-related skin conditions. In this study, we evaluated pHCA (one of the most abundant naturally occurring hydroxycinnamic acid metabolites [11]), on stress-induced cellular responses in human skin models. We specifically tested its impact on cellular senescence and epidermal homeostasis, inflammatory SASP molecules, DNA damage, and energy metabolism, in a combination of 2D cultures of HPKs, immortalized keratinocytes, and 3D organotypic skin models. Here we show that pHCA significantly prevents oxidative stress-induced cellular senescence in both 2D HPK cultures and 3D organotypic epidermal models. In 2D models, a pre-stress treatment with pHCA significantly reduces 53BP1 DNA repair foci in H_2_O_2_-stressed keratinocytes, indicating the prevention of DNA damage (presumably via an antioxidation mechanism). Importantly, the positive NAM control showed an increase in 53BP1 foci number, especially when used as a post-treatment, confirming the improved DNA damage repair in the presence of NAM, as previously reported [5]. In 3D organotypic models, we observed that pHCA had a directionally higher anti-inflammatory effect at reducing IL-6 and IL-8 than the NAM positive control. This is intriguing given that both pHCA and NAM attenuated senescence (measured by lamin B1 and p21 levels) within similar orders of magnitude. The pHCA treatment was also found to reduce PGE_2_, known to be upregulated by the NF-κB pathway after stress in keratinocytes, potentially explaining this difference, and supporting a broader anti-inflammatory effect of pHCA beyond senescence reduction [27]. By contrast, we found that NAM had a stronger effect than pHCA on rescuing epidermal homeostasis with reduced stress-induced differentiation. This was consistently observed in both 2D and 3D models and is probably linked to the increased metabolism from NAD^+^ pools present in basal stem cells in the presence of NAM, but not pHCA. Lastly, since mitochondrial dysfunction has been shown to drive senescence (known as mitochondrial dysfunction-associated senescence—MiDas) [28], and since ROS drives mitochondrial dysfunction [29,30,31,32], we investigated the effect of pHCA on the mitochondrial function of hTERT keratinocytes using metabolic stress assays. pHCA was also assessed under stress conditions for both ATP production and glycolytic stress. pHCA treatment demonstrated significant improvements of respiration and ATP production, as well as better glycolytic capacity and reserve, which may aid reducing stress-induced senescence, along with the other DNA damage mitigation and anti-inflammatory mechanisms previously reported.

Regarding mechanisms, senescence and the associated inflammation can be regulated via actives that either prevent senescence itself (such as NAM and rapamycin), suppress the SASP (i.e., senomorphics, e.g., flavonoids such as fisetin), or by senolytics which selectively kill senescent cells (such as quercetin, dasatinib, and navitoclax) [33]. In skin, we have shown that NAM prevents senescence and inflammation through the maintenance of energy metabolism and improved DNA repair [5], while rapamycin via suppression of the mTOR pathway (known to be activated in senescent cells) reduces p16 expression, improves the organization of the epidermis basal layer, and increases collagen VII levels and dermal thickness, eventually leading to a decrease in fine wrinkles and sagging [34]. Fisetin was shown to increase filaggrin expression and reduce transepidermal water loss (TEWL), improving barrier function. In addition, fisetin reduces MMP-1 and MMP-2 expression, while also limiting inflammation (notably PGE_2_, IL-6, and COX-2 expression) through the upregulation of Nrf2, known to modulate the transcriptional activation of antioxidant genes by binding to ARE (antioxidant response elements) present in their promoter region [35,36,37]. Nrf2 can be activated by xenobiotics such as 2,3,7,8-tetrachlorodibenzo-*p*-dioxin (TCDD) via the binding of TCDD to the aryl hydrocarbon receptor (AhR), followed by nuclear translocation and heterodimerization with the AhR nuclear translocator protein (ARNT), and further binding to the XREL (xenobiotic response element (XRE)-like element) present in the *Nrf2* promoter [38]. Importantly, AhR is gaining recognition as a significant immune modulator, known to bind naturally occurring phytochemicals, such as flavonoids [39]. Consequently, a possibility that could explain the strong effect of pHCA on inflammation observed here is a potential binding of pHCA to AhR, followed by the modulation of *IL-6*, *IL-8*, and *PGE_2_* expression. Alternatively, *Nrf2* mRNA levels could be increased via pHCA regulation of the NF-κB, KRAS, BRAF, MYC, Notch, or PI3K-AKT pathways, all of which have been shown to regulate *Nrf2* transcription [40,41,42,43].

Surprisingly, pHCA demonstrated significant anti-senescence properties when used both as a pre- and post-stress treatment. If for the pre-stress treatment, senescence inhibition can be attributed to the prevention of DNA damage by pHCA (by opposition to other compounds of the same family which improve DNA repair), in the post-stress treatment scenario, additional mechanisms are certainly involved since DNA damage is not prevented. The stoichiometry of the H_2_O_2_ stressor (125 μM) used in 3D culture was significantly higher than the pHCA (10 μM), suggesting that a direct free radical or ROS quenching mechanism alone cannot explain the effects observed either. We hypothesize that the inhibition of senescence in the post-stress treatment protocol could be related to the modifications of mitochondrial function observed here. However, this assumption requires further investigation. In addition, more work is needed to clarify how pHCA specifically prevents DNA damage (that could go beyond the prevention of ROS generation), as well as to elucidate the pathways underlying its very potent anti-inflammatory effects, which likely extend past senescence inhibition, and could involve the AhR pathway as mentioned above.

Lastly, and moving forward, these results are in favor of potential complementary treatments of different chemistries working on separate pathways. Specifically, pHCA and NAM could be tested in combination, where pHCA would prevent DNA damage while NAM would improve DNA repair and inhibit premature differentiation [5]. Likewise, their effects on inflammation and mitochondrial function could be synergistic, overall improving the anti-aging effects of both molecules used individually. Further work is needed to continue to investigate the molecular mechanisms associated with pHCA; however, based on the results presented here, there is substantial evidence that pHCA, alone or in combination with other compounds, can be an effective treatment to prevent the signs of premature skin aging.

## 4. Materials and Methods

### 4.1. 2D Cell Culture and Stress Treatments

HPKs were obtained from healthy human skin samples (6-year-old male donor, foreskin) from de-identified surplus surgical waste with written informed patient consent and ethical clearance. The usage of HPKs was approved by the A*STAR Institutional Review Board 2020-209.

HPKs were isolated as previously described [44]. For maintenance, HPKs were cultured on lethally irradiated murine 3T3-J2 feeder cells in cFAD medium (3:1 Dulbecco’s Modified Eagle Medium (DMEM)/Ham’s F-12) supplemented with 10% fetal calf serum, 1% penicillin/streptomycin, and 10 ng/mL epidermal growth factor, as previously described [45,46,47]. The medium was replaced every 2–3 days. One day before the commencement of stress experiments, HPKs were seeded in Dermalife^®^ medium (feeder-free system, LL-0007; Lifeline Cell Technology, Oceanside, CA, USA). For all experiments conducted with H_2_O_2_ treatment, except DNA repair experiments, HPKs were treated with 63 µM H_2_O_2_ ± pHCA at the indicated concentrations or ±1.5 mM NAM for 30 min, and the medium was replaced immediately after H_2_O_2_ exposure. Cells were re-fed ± pHCA or NAM every 2–3 days and analyzed 5 days after H_2_O_2_ treatment. For DNA repair analyses, HPKs were either pre-treated with pHCA or NAM for 24 h before H_2_O_2_ challenge (pHCA and NAM were not added during the H_2_O_2_ treatment, nor after), or treated with pHCA or NAM during the H_2_O_2_ challenge, followed by fresh medium change with pHCA or NAM addition. Cells were fixed immediately after H_2_O_2_ treatment, and 15 min, 4 h, and 24 h after H_2_O_2_ treatment.

hTERT keratinocytes were obtained as a kind gift from Dr. Jerry Shay, University Texas Southwestern. They were expanded at 37 °C with 5% CO_2_ using the Epilife^TM^ medium (ThermoFisher Scientific, Waltham, MA, USA) with human keratinocyte growth supplement, and gentamicin/amphotericin B.

HaCaT cells (a generous gift from Dr. Fiona Watt, King’s College London, London, UK) were cultured at 37 °C with 5% CO_2_ in DMEM supplemented with 10% fetal bovine serum, penicillin/streptomycin 100 units/mL, and glutamax 100 units/mL.

### 4.2. Clonogenicity Assays and Rhodamine B Staining

After the completion of the H_2_O_2_ treatment ± pHCA or NAM experiment, clonogenicity tests were performed by seeding either 750 or 1200 HPKs on 3T3-J2 feeder cells on 10 cm Petri dishes. HPKs were grown for 12 days before Rhodamine B staining and the quantification of colony number and area (Supplementary Materials and Methods). For colony visualization, dishes were treated with 3.7% formaldehyde/phosphate buffered saline (PBS, Merck, Rahway, NJ, USA) for 15 min, before staining for 4–6 h with 1% Rhodamine B (J.T Baker^®^, Phillipsburg, NJ, USA) dissolved in water. Dishes were washed 3 times with water before colony analyses.

### 4.3. Reverse Transcription-Quantitative Polymerase Chain Reaction (RT-qPCR)

Total mRNA was extracted, and reverse transcribed as previously described [47]. SYBR green RT-qPCR was carried out using the primer sequences listed in Supplementary Table S1.

### 4.4. Protein Extraction and Western Blotting

Proteins extraction and Western blotting were conducted as previously described [44], using the specific antibodies listed in Supplementary Table S2.

### 4.5. 53BP1 Staining and Quantification

HPKs were fixed in 2% paraformaldehyde at 4 °C for 30 min and treated with 2 M hydrogen chloride for 30 min. Cells were then blocked with 20% fetal bovine serum followed by a 30 min incubation with primary antibodies (Supplementary Table S2) at 37 °C. Secondary detection was achieved with an Alexa Fluor 568 antibody (Invitrogen, Carlsbad, CA, USA), and Hoechst (Invitrogen) was used as a nuclear dye. Coverslips were mounted onto slides using ProLong Diamond Antifade Mountant (ThermoFisher Scientific), and images were acquired on an IX-83 Olympus (Evident Corporation, Tokyo, Japan) microscope. The number of 53BP1 foci per cell was quantified using ImageJ version 1.52o (National Institutes of Health, Bethesda, MD, USA), as previously described [48].

### 4.6. 3D Human Skin Organotypic Models

EpiDerm^TM^ human epidermis models (EPI-200-3S, MatTek Corporation, Ashland, MA, USA) were used for the H_2_O_2_ challenge experiments. Tissues were cultured and air-lifted according to the manufacturer’s protocol. At the completion of stratification, tissues were pre-treated with 10 µM pHCA for 16 h prior to a 30 min challenge with 125 µM H_2_O_2_ (added to the medium). The medium was replaced immediately after H_2_O_2_ treatment by fresh medium without pHCA, and cells were re-fed every 2 days. For post-stress treatments, 10 µM pHCA or 1.5 mM NAM were added immediately after H_2_O_2_ challenge, and cells were re-fed every 2 days with medium containing pHCA or NAM. Tissues were harvested on day 6 after H_2_O_2_ treatment. The samples were fixed in 10% neutral buffered formalin for 4 h at room temperature followed by paraffin-embedded preparation, as previously described [47].

EpiDermFT^TM^ human full-thickness skin models (EFT-400-7A, MatTek Corporation) were used for the UV challenge experiments. Tissues were received and acclimatized for 4 h prior to the commencement of 50 µM pHCA or 1.5 mM NAM treatment. After a 2-day pre-treatment, tissues were irradiated with 1.7 J/cm^2^ UVA + 25 mJ/cm^2^ UVB using a BioSun (Vilber, Collégien, France). Supernatants were collected 48 h after UV exposure, and IL-6 and IL-8 levels were quantified using enzyme-linked immunosorbent assays (ELISAs).

### 4.7. Enzyme-Linked Immunosorbent Assay (ELISA) in 3D Organotypic Cultures

ELISAs were performed on culture supernatants derived from 3D full-thickness models. Quantitative measurements of IL-6 and IL-8 were conducted according to the manufacturer’s instructions (antibodies are listed in Supplementary Table S2), using a microplate reader (Spark, Tecan, Männedorf, Switzerland). A quantitative analysis of samples was performed using a Four Parameter Logistic curve fit (https://www.myassays.com/index.html).

### 4.8. PGE_2_, IL-6, and IL-8 Measurements in 2D Cultures

For PGE_2_ measurements, 2.5 μg/mL arachidonic acid was supplemented into the hTERT culture medium. A homogeneous time resolved fluorescence (HTFR) kit (CisBio, Bedford, MA, USA) was used to measure PGE_2_ from the culture supernatants, and data were normalized to the ATP values derived from the same cells. ATP was quantified using the CellTiter-Glo^®^ assay (Promega, Madison, WI, USA). The media was removed, cells were washed with 200 μL of PBS, and 100 μL of medium was added to each well. The CellTiter-Glo^®^ reagent was prepared according to the manufacturer’s instructions and 100 µL were added to the cells. Cells were incubated with reagent as per the manufacturer’s instructions, and 200 µL were transferred from the 24 well V7 plate to a black clear bottom 96-well plate to quantify ATP via luminescence (BioTek Synergy plate reader, Agilent Technologies, Santa Clara, CA, USA).

IL-8 and IL-6 were quantified using meso scale discovery (MSD) electrochemiluminescence V-Plex cytokine kits, as per the manufacturer’s instructions (Meso Scale Diagnostics, Rockville, MD, USA), and normalized to the amount of soluble total proteins as determined by bicinchoninic acid protein assays (BCA™ Protein Assay Kit, Pierce Biotechnology/Thermo Scientific, Rockford, IL, USA).

### 4.9. Immunofluorescence Staining of 3D Organotypic Sections

Paraffin-embedded sections of 4 µm thickness were prepared and stained for differentiation and senescence markers, as previously described [5]. In brief, tissue sections were dewaxed, rehydrated, and underwent heat-induced antigen retrieval (2100 antigen retriever, Aptum Biologics, UK). Sections were then blocked for 30 min with 10% goat serum followed by incubation for 1 h with primary antibodies (Supplementary Table S2). Secondary antibodies (Alexa Fluor 488 or 568, Invitrogen) were used to detect the targets, and nuclei were counterstained with DAPI (Sigma-Aldrich, St. Louis, MO, USA) before mounting with Hydromount (Electron Microscopy Sciences, Hatfield, PA, USA).

### 4.10. Lamin B1 Quantification

Images were acquired on a Nikon Eclipse Ni-E upright microscope (Nikon, Tokyo, Japan). Perinuclear lamin B1 expression levels were quantified in each individual cell using ImageJ version 1.52o (National Institutes of Health) with a self-composed macro (Supplementary Materials and Methods, and [5,49,50]), and protein levels per cell were reported on dot plots for both the basal (K10-negative) and upper (K10-positive) layers.

### 4.11. Seahorse Metabolic Stress Assays

Metabolic stress testing was conducted from hTERT immortalized keratinocytes using the Seahorse XFe FluxAnalyzer™ (Agilent Technologies). Mitochondrial function was assessed with Mito Stress assays using oligomycin, FCCP, rotenone, and antimycin A at final concentrations of 0.5 μg/mL, 0.5 μM, 0.25 μM, and 0.25 μM, respectively. Glycolytic function was assessed by Glyco Stress assays using glucose, oligomycin, and 2-deoxy-D-glucose at final concentrations of 1 mM, 1 μM, and 5 mM, respectively. To allow comparison between different experiments, data were expressed as the rate of oxygen consumption in pmol/minute or the rate of extracellular acidification in mpH/minute, normalized to cell protein in individual wells determined by a BCA protein assay (Pierce Biotechnology/Thermo Scientific).

### 4.12. CellROX Assays

A 60 min pHCA treatment was applied to HaCaT keratinocytes, diluted in standard DMEM. Following treatment, cells were washed 3 times with PBS and CellROX Deep Red (ThermoFisher Scientific) was added at a concentration of 5 μM. After 30 min, cells were washed 3 times with PBS, and Fluorobrite media (ThermoFisher Scientific) was added prior to imaging. The ROS fluorescence images were acquired with a Zeiss LSM 880 with Airyscan^®^ (Zeiss, Oberkochen, Germany) using Zeiss Zen 2.3 SP1 FP3 (Black) (Zeiss), to visualize the spatial distribution of ROS within the cells. Images were quantified in Fiji version 2.14.0/1.54f (National Institutes of Health), using the integrated density for the total field of view given equivalent cell confluency across experiments, to determine the corrected total fluorescence values. The integrated density is the sum of the values of the fluorescence pixel intensity corrected for the background level of fluorescence.

### 4.13. Statistical Analyses

Statistical analyses were performed using GraphPad Prism, version 9 (GraphPad software, San Diego, CA, USA), with Student’s *t*-tests or one-way or two-way ANOVA as indicated. *p* ≤ 0.05 indicates statistical significance. (* *p* ≤ 0.05; ** *p* ≤ 0.01; *** *p* ≤ 0.001; **** *p* ≤ 0.0001). All data represent the means ± SEM (standard error of the mean), except for the Seahorse experiments where data represent the means ± SD (standard deviation).

## Data Availability

The original contributions presented in the study are included in the article/supplementary materials, further inquiries can be directed to the corresponding authors.

## Supplementary Materials

The following supporting information can be downloaded at: https://www.mdpi.com/article/10.3390/ijms25158153/s1. Reference [51] is cited in the supplementary materials.
