# Supplementary material for: Para-Hydroxycinnamic Acid Mitigates Senescence and Inflammaging in Human Skin Models
Source: Int J Mol Sci. 2024 Jul 26;25(15):8153. doi: 10.3390/ijms25158153 (PMC11312399; doi:10.3390/ijms25158153)
Supplement: Supplementary file 1 [file ijms-25-08153-s001.zip › Supplementary Materials-rev3.pdf]

LEGENDS TO SUPPLEMENTARY FIGURES

Supplementary Figure S1: Analyses of proliferation in 3D organotypics after UVB irradiation +/- pHCA. (**A**) Percentage of Ki-67–positive cells in the whole epidermis. (**B**) Percentage of Ki-67–positive cells in the basal layer only. A total of 16-20 fields per condition were analyzed (from triplicates). Statistical analyses: one-way ANOVA, n = 3. Abbreviations: pHCA, *para*-hydroxycinnamic acid; NAM, niacinamide; n.s., not significant.

Supplementary Figure S2: pHCA used as a pre-stress treatment prevents H_2O_2-induced DNA damage. Example of immunofluorescence images (630x) used for the quantification of 53BP1 foci shown in Figure 5A. Scale bar: 50 µm. For each picture, a magnification is shown (white rectangle). Abbreviations: pHCA, *para*-hydroxycinnamic acid; NAM, niacinamide.

Supplementary Figure S3: pHCA used as a post-stress treatment does not prevent H_2O_2-induced DNA damage and does not improve DNA repair. Example of immunofluorescence (630x) images used for the quantification of 53BP1 foci shown in Figure 5B. Scale bar: 50 µm. For each picture, a magnification is shown (white rectangle). Abbreviations: pHCA, *para*-hydroxycinnamic acid; NAM, niacinamide.

SUPPLEMENTARY TABLES

Supplementary Table S1: Primer sequences used for RT-qPCR

Primer name	Sequence (5′–3′)
TBP	F: CGTCCCAGCAGGCAACAC<br>R: TTGTGAGAGTCTGTGAGTGGAAGAG
Involucrin	F: CCATCAGGAGCAAATGAAACAG<br>R: GCTCGACAGGCACCTTCTG
Filaggrin	F: GTGTTAGTTACAATTCCAATCCTGTTG<br>R: ATACGTTGCATAATACCTTGGATGATC
Lamin B1	F: CGCTTGGTAGAGGTGGATTCTG<br>R: CCTCACTTGGGCATCATGTTG
p21	F: GCGACTGTGATGCGCTAATG<br>R: CGGTGACAAAGTCGAAGTTCC

Abbreviations: F: Forward; R: Reverse, TBP: TATA-binding protein

Supplementary Table S2: Primary antibodies used for 2D immunofluorescence, Western blotting, 3D immunofluorescence and ELISA

Primary antibody	Dilution	Source (catalogue number; supplier)
2D Immunofluorescence		
53BP1	1:3000	NB100-304; Novus Biologicals, CO, USA
Western blotting		
Actin	1:1000	A2066; Sigma–Aldrich, MO, USA
Involucrin	1:500	Novocastra NCL-INV; Leica Biosystems, IL, USA
Lamin B1	1:10000	ab16048; Abcam, Cambridge, UK
p21	1:500	Waf1/Cip1 (12D1), 2947; Cell Signaling, MA, USA
3D Immunohistochemistry/fluorescence		
K10	1:400	DE-K10, M7002, DAKO, Agilent, CA, USA
Lamin B1	1:1000	ab16048, Abcam, Cambridge, UK
Ki-67	1:200	MIB-1; M7240; Dako, Agilent, Santa Clara, CA, USA
ELISA		
IL-6	-	88-7066-86, Invitrogen, MA, USA
IL-8	-	431504, BioLegend, CA, USA

SUPPLEMENTARY MATERIALS AND METHODS

Macro used for quantification of Lamin B1

```
//John Lim, AMP, June 2022

//Segment nuclei using Machine Learning plugin LabKit

//Please cite LabKit: Arzt, M., Deschamps, J., Schmied, C., Pietzsch, T., Schmidt, D., Tomancak, P., ... Jug, F. (2022). LABKIT: Labeling and Segmentation Toolkit for Big Image Data. Frontiers in Computer Science, 4. doi:10.3389/fcomp.2022.777728

//Macro will run auto background substraction based on rolling ball radius

//Macro is setup for batch processing


//Source folder

classifier = "06062022_nuclei.classifier"; //filename for classifier used by LabKit

image_ext = ".nd2"; //input image file extension


//Channels & area (follow unit of raw image)

nuclei_ch = 3;

minSize = 3.6; //nuclei minimum area

maxSize = 250; //cluster of nuclei maximum area

max_ch = 1; //channel for mean intensity

max_ch_bg = 560;

threshold_mean_ch = 2; //channel for threshold mean intensity, brighter pixels

threshold_mean_ch_bg = 760;


//substrate min area (follow unit of raw image)

substrate = 1400;


//input folder

inDir = getDirectory("Select input folder");

files = getFileList(inDir);

len = files.length;
```

```
//table
Table.create("Data_Table");
Table.showRowNumbers(true);
Table.setLocationAndSize(0, 0, 600, 600);

//main
requires("1.52a"); //check ImageJ version
setOption("BlackBackground", true);
roiManager("reset");
//setBatchMode(true);

for (i = 0; i < len; i++) {
	if(endsWith(files[i], image_ext) == true){
		roiManager("reset");
		run("Clear Results");
		close("*");

		run("Bio-Formats Importer", "open=["+inDir+files[i]+"] autoscale color_mode=Default rois_import=[ROI manager] view=Hyperstack stack_order=XYCZT");
		id0 = getImageID();
		title0 = getTitle();
		getPixelSize(unit, pixelWidth, pixelHeight);

		//Labkit
		selectImage(id0);
		run("Duplicate...", "title=nuclei_Labkit duplicate channels=nuclei_ch");
		id1 = getImageID();
		run("Segment Image With Labkit", "input=[nuclei_Labkit] segmenter_file=["+inDir+classifier+"] use_gpu=false");
		setOption("ScaleConversions", true);
		run("8-bit");
```

```
run("Set Scale...", "distance=1 known=0.1833741 unit=micron");
id2 = getImageID(); //image id for output image

//substrate
selectImage(id2);
setAutoThreshold("Default dark");
run("Analyze Particles...", "size="+substrate+"-Infinity circularity=0.00-1.00 add");
sCount = roiManager("count");
if(sCount != 1){
	print("More than one or Zero substrate was detected "+title0);
	substrate_y = "NA";
}
if(sCount == 1){
	roiManager("select", 0);
	List.setMeasurements();
	substrate_y = List.getValue("Y");
	//roiManager("reset");
}

//nuclei
selectImage(id2);
run("Select None");
roiManager("reset");
setAutoThreshold("Default dark");
run("Analyze Particles...", "size="+minSize+"-"+maxSize+" circularity=0.00-1.00 show=Masks");
id3 = getImageID(); //image id for size selected nuclei image
run("Grays");
run("Fill Holes");
run("Watershed");
run("Analyze Particles...", "size="+minSize+"-"+maxSize+" circularity=0.25-1.00 show=Nothing exclude add");
```

```
nROI = roiManager("count");

//intensity measurement
if(nROI > 0){ //do intensity measurement if there is nucleus
	for (j = 0; j < nROI; j++) { //loop through nuclei
		selectImage(id0);
		Stack.setChannel(max_ch);
		resetThreshold();
		roiManager("select", j);
		roiName = Roi.getName;
		List.setMeasurements();
		x = List.getValue("X");
		y = List.getValue("Y");
		area = List.getValue("Area");
		max1 = List.getValue("Max");
		max1 = max1 - max_ch_bg;
		Stack.setChannel(threshold_mean_ch);
		setAutoThreshold("Default dark");
		getThreshold(lower, upper);
		setThreshold(lower, upper);
		List.setMeasurements("limit");
		mean1 = List.getValue("Mean");
		mean1 = mean1 - threshold_mean_ch_bg;
		if(substrate_y == "NA"){
			ydistanceS = "NA";
		}
		else{
			ydistanceS = substrate_y - y;
		}
		//output to table
		nrow = Table.size; //print(nrow);
```

```
                    Table.set("Label", nrow, title0+"_"+roiName);
                    Table.set("X", nrow, x); Table.set("Y", nrow, y);
                    Table.set("Area", nrow, area);
                    Table.set("Max_Intensity", nrow, max1);
                    Table.set("Threshold_MeanIntensity", nrow, mean1);
                    Table.set("ydistanceS", nrow, ydistanceS);
                    Table.update;
                }
                roiManager("Deselect");
                roiManager("Show All without labels");
                roiManager("save", inDir+title0+"_ROIs.zip"); //save ROIs set
            }


    }
}

print("Done"); //end of macro
```

Macro for colony number and area analyses

```
//John Lim, AMP
//Custom Colonies counting, must use clear plate with no background pattern
//Uses Colour Deconvolution to segment the colonies

mod = 0.3; //modifier for threshold, range from 0.1 to 2, default is 1

//Dialog box
Dialog.create("Custom colonies plate counting");
Dialog.addMessage("By John Lim, john0limsy@gmail.com, AMP");
```

```
Dialog.addMessage("No background pattern on plate");
Dialog.addMessage("RGB images white background");
Dialog.addMessage("Uses Colour Deconvolution with");
Dialog.addMessage("3 sets of predetermined values");
Dialog.addNumber("Exclude edge (pixel)", 150); //exclusion zone, exclude things near the edge in this zone around edge
Dialog.addNumber("Calibration (pixel per mm)", 18.30); //number of pixels per mm
Dialog.addNumber("Plate area (pixel)", 755000); //estimated minimum size (pixels) of plate
Dialog.addMessage("Colony size range (pixel)");
Dialog.addNumber("Smallest_colony", 100);
Dialog.addNumber("Largest_colony", 50000);
Dialog.addCheckbox("Run as batch processing", false); //set to true for batch processing
Dialog.addString("Extension for images for batch processing", ".JPG");
//Dialog.show();


//Variables
exclude = Dialog.getNumber(); //thickness of plate to exclude from analysis
calibration = Dialog.getNumber();
plate_area = Dialog.getNumber();
min_size = Dialog.getNumber(); //colony
max_size = Dialog.getNumber(); //colony
//batch = false;
batch = Dialog.getCheckbox();
ext = Dialog.getString();


//Predetermined values for Colour Deconvolution
//[r1]=0.4994966 [g1]=0.6900051 [b1]=0.52382827 [r2]=0.5413001 [g2]=0.6223186 [b2]=0.5654323 [r3]=0.5529624 [g3]=0.63206625 [b3]=0.54288566
a1=0.4994966; a2=0.6900051; a3=0.52382827;
b1=0.5413001; b2=0.6223186; b3=0.5654323;
c1=0.5529624; c2=0.63206625; c3=0.54288566;
```

```
//setup
run("Set Measurements...", "area display redirect=None decimal=3");
setOption("BlackBackground", true);
roiManager("reset");
run("Clear Results");
setForegroundColor(255, 255, 255);
setBackgroundColor(0, 0, 0);
setBatchMode(true);

//Batch
if(batch == true){
	inDir = getDirectory("Choose input folder");
	files = getFileList(inDir);
	len = files.length; //print(len);
}
else{
	len = 1;
	skip = false;
}

//Main
for(j=0; j<len; j++){
	if(batch == true){
		run("Clear Results");
		roiManager("reset");
		run("Close All");
		if(endsWith(files[j], ext)){ //if(endsWith(files[j], ".tif") || endsWith(files[j], ".jpg")){
			open(inDir+files[j]);
			skip = false;
		}
		else{
```

```
			print(files[j]+" is not a .tif or .jpg");
			skip = true;
		}
	}

	if(skip == false){
		name0 = getTitle();
		id0 = getImageID();
		run("Set Scale...", "distance="+calibration+" known=1 unit=mm");

		//find plate
		selectImage(id0);
		run("Select None");
		run("Duplicate...", "title="+name0+"_plate");
		id1 = getImageID();
		run("8-bit");
		run("Gaussian Blur...", "sigma=1");
		run("Find Edges");
		setAutoThreshold("Triangle dark");
		run("Analyze Particles...", "size="+plate_area+"-Infinity pixel circularity=0.10-1.00
include add");
		if(roiManager("count")!=1){
			//exit("A single plate was not detect");
			print("Single plate was not detected for "+name0);
		}
		else{
			//Colour Deconvolution
			selectImage(id0);
			run("Select None");
			run("Duplicate...", "title="+name0+"_ColDec");
			id2 = getImageID();
```

```
title2 = getTitle();
run("Colour Deconvolution", "vectors=[User values] [r1]=0.4994966 [g1]=0.6900051 [b1]=0.52382827 [r2]=0.5413001 [g2]=0.6223186 [b2]=0.5654323 [r3]=0.5529624 [g3]=0.63206625 [b3]=0.54288566 hide");
selectWindow(title2+"-(Colour_2)"); close();
selectWindow(title2+"-(Colour_3)"); close();
selectImage(id1); close();
selectImage(id2); close();


//Colonies
selectWindow(title2+"-(Colour_1)");
run("Set Scale...", "distance="+calibration+" known=1 unit=mm");
id3 = getImageID();
roiManager("Select", 0);
setAutoThreshold("Default");
getThreshold(lower, upper);
setThreshold(lower, upper*mod);
run("Convert to Mask");
run("Grays");
run("Fill Holes");
run("Watershed");
roiManager("Select", 0);
run("Enlarge...", "enlarge=-"+exclude+" pixel");
roiManager("Add");
roiManager("Select", 1);
run("Analyze Particles...", "size="+min_size+"-"+max_size+" pixel circularity=0.20-1.00 summarize add");
nROI = roiManager("count");
selection = newArray(nROI-2);
for(i=2; i<nROI; i++){
	selection[i-2] = i;
}
```

```
                roiManager("select", selection);
                roiManager("Measure");
                selectImage(id3); close();
                selectImage(id0);
                roiManager("Show All without labels");
                roiManager("Deselect");

                if(batch == true){
                    //save ROI
                    roiManager("Save", inDir+name0+"_RoiSet.zip");
                    //save Results
                    selectWindow("Results");
                    saveAs("Results", inDir+name0+"_Results.csv");
                }

            }
        }
}
```
