# Supplementary figures and images for: Para-Hydroxycinnamic Acid Mitigates Senescence and Inflammaging in Human Skin Models
Source: Int J Mol Sci. 2024 Jul 26;25(15):8153. doi: 10.3390/ijms25158153 (PMC11312399; doi:10.3390/ijms25158153)

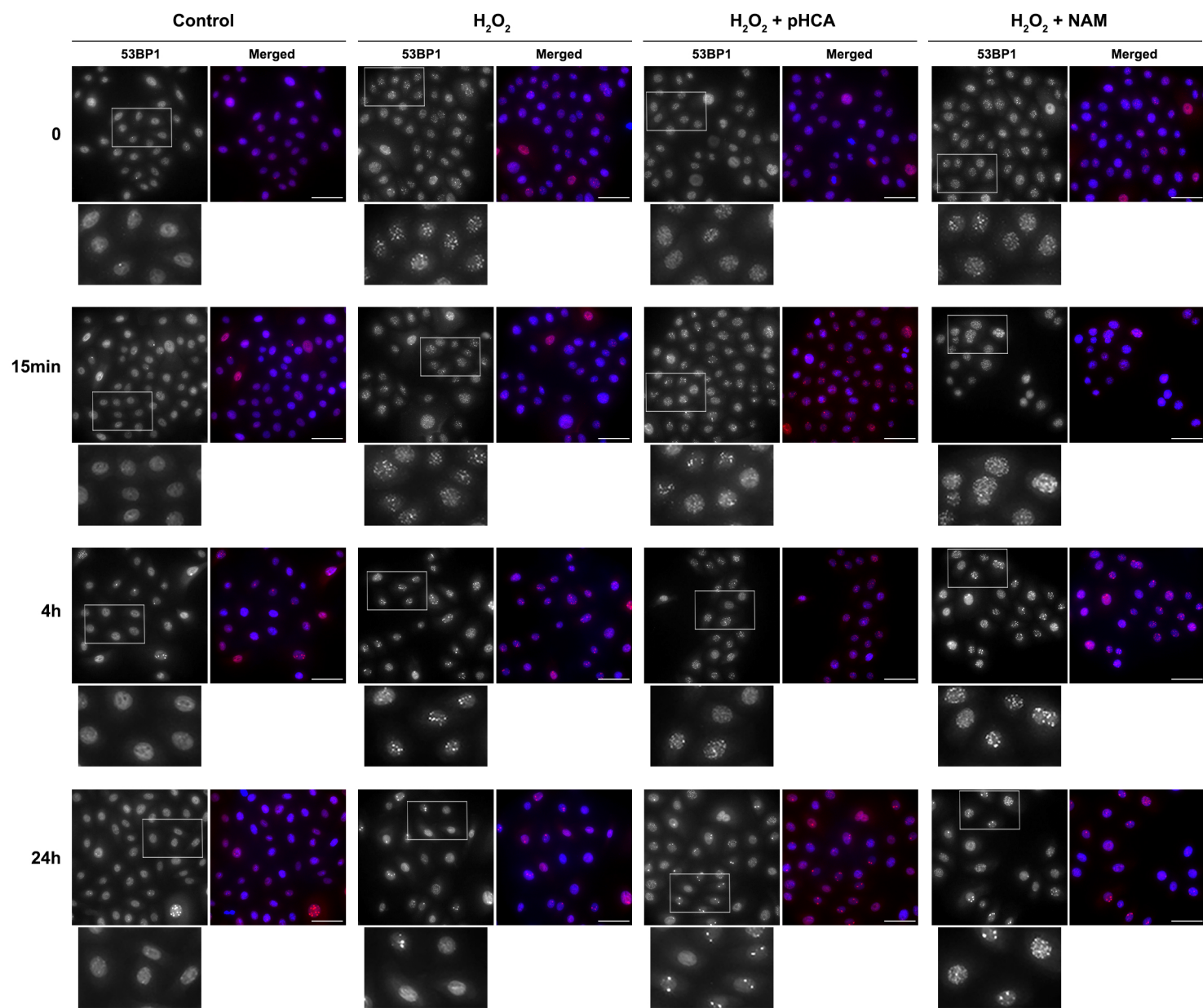

Supplement: Supplementary file 1 [file ijms-25-08153-s001.zip › Supplementary Figure S3-rev3.pdf]

**A**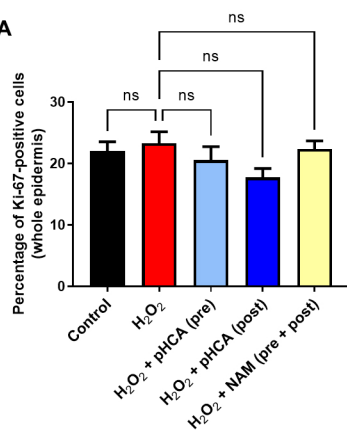**B**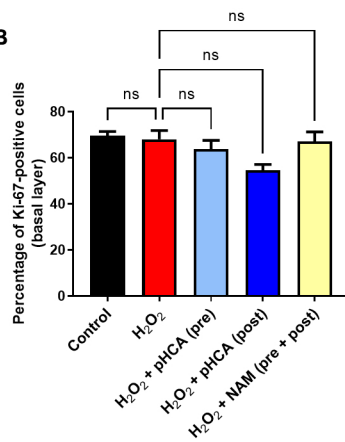

Supplement: Supplementary file 1 [file ijms-25-08153-s001.zip › Supplementary Figure-S1-rev3.pdf]

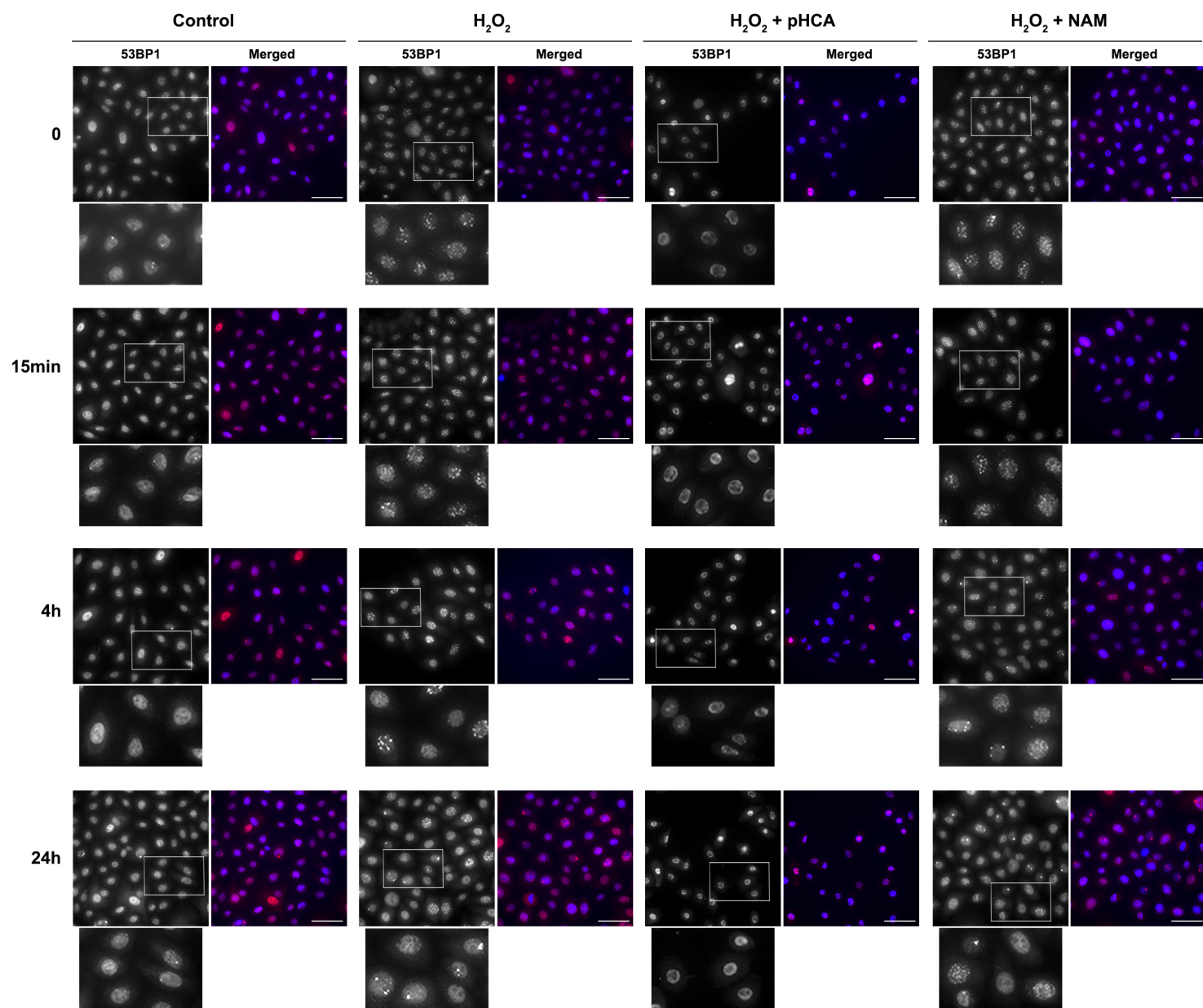

Supplement: Supplementary file 1 [file ijms-25-08153-s001.zip › Supplementary Figure-S2-rev3.pdf]
